# Prevalence of diagnostically-discrepant *Clostridioides difficile* clinical specimens: insights from longitudinal surveillance

**DOI:** 10.3389/fmed.2023.1238159

**Published:** 2023-10-18

**Authors:** Farhan Anwar, Marielle Clark, Jason Lindsey, Rachel Claus-Walker, Asad Mansoor, Evy Nguyen, Justin Billy, William Lainhart, Kareem Shehab, V. K. Viswanathan, Gayatri Vedantam

**Affiliations:** ^1^School of Animal and Comparative Biomedical Sciences, University of Arizona, Tucson, AZ, United States; ^2^Department of Pathology, Clinical Microbiology Laboratories, Banner University Medical Center, Tucson, AZ, United States; ^3^Department of Pediatrics, College of Medicine, University of Arizona, Tucson, AZ, United States; ^4^Bio5 Institute for Collaborative Research, University of Arizona, Tucson, AZ, United States; ^5^Southern Arizona VA Healthcare System, Tucson, AZ, United States

**Keywords:** *Clostridioides difficile*, prevalence, surveillance, ribotyping, discrepant, longitudinal

## Abstract

**Background:**

*Clostridioides difficile* Infection (CDI) is a healthcare-associated diarrheal disease prevalent worldwide. A common diagnostic algorithm relies on a two-step protocol that employs stool enzyme immunoassays (EIAs) to detect the pathogen, and its toxins, respectively. Active CDI is deemed less likely when the Toxin EIA result is negative, even if the pathogen-specific EIA is positive for *C. difficile.* We recently reported, however, that low-toxin-producing *C. difficile* strains recovered from Toxin-negative (‘discrepant’) clinical stool specimens can be fully pathogenic, and cause lethality in a rodent CDI model. To document frequency of discrepant CDI specimens, and evaluate *C. difficile* strain diversity, we performed longitudinal surveillance at a Southern Arizona tertiary-care hospital.

**Methods:**

Diarrheic stool specimens from patients with clinical suspicion of CDI were obtained over an eight-year period (2015–2022) from all inpatient and outpatient Units of a > 600-bed Medical Center in Southern Arizona. Clinical laboratory EIA testing identified *C. difficile*-containing specimens, and classified them as Toxin-positive or Toxin-negative. *C. difficile* isolates recovered from the stool specimens were DNA fingerprinted using an international phylogenetic lineage assignment system (“ribotyping”). For select isolates, toxin abundance in stationary phase supernatants of pure cultures was quantified via EIA.

**Results:**

Of 8,910 diarrheic specimens that underwent diagnostic testing, 1733 (19.4%) harbored *C. difficile*. Our major findings were that: (1) *C. difficile* prevalence and phylogenetic diversity was stable over the 8-year period; (2) toxigenic *C. difficile* was recovered from 69% of clinically Tox-neg (‘discrepant’) specimens; (3) the six most prevalent USA ribotypes were recovered in significant proportions (>60%) from Tox-neg specimens; and (4) toxin–producing *C. difficile* recovered from discrepant specimens produced less toxin than strains of the same ribotype isolated from non-discrepant specimens.

**Conclusion:**

Our study highlights the dominance of Toxin EIA-negative CDI specimens in a clinical setting and the high frequency of known virulent ribotypes in these specimens. Therefore, a careful reevaluation of the clinical relevance of diagnostically-discrepant specimens particularly in the context of missed CDI diagnoses and *C. difficile* persistence, is warranted.

## Graphical abstract

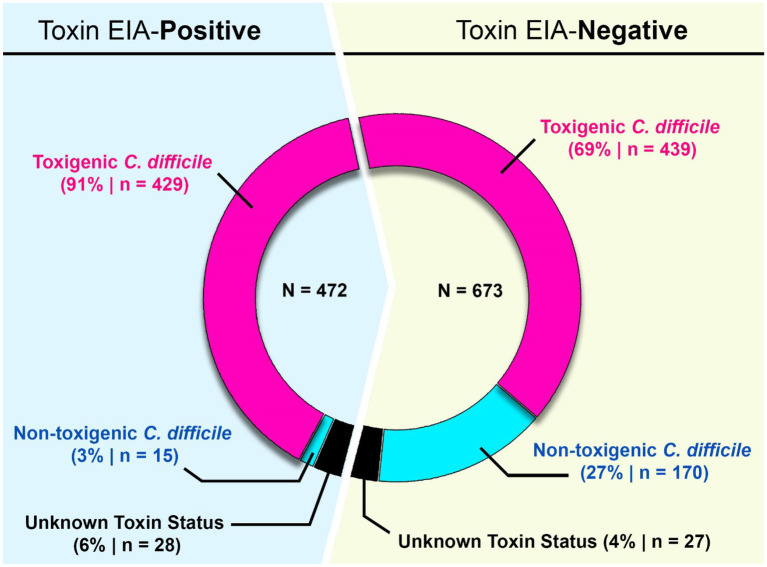

## Introduction

*Clostridioides difficile* is a Gram-positive, spore-forming bacillus that can produce up to three toxins that potentiate gastrointestinal disease, typically antibiotic-associated, with symptoms ranging from mild–moderate diarrhea to pseudomembranous colitis (1, 2). In the United States, there are over 223,000 cases of *C. difficile* infection (CDI) annually resulting in an economic burden exceeding $1 billion; therefore, CDI is also one of the most prevalent healthcare-associated infections (HAIs) in the USA (3). Certain phylogenetic lineages of *C. difficile* (ribotypes; RTs), such as RT027 have been associated with higher rates of recurrent infection, greater incidence of severe disease, antibiotic resistance, and more frequent outbreaks (4). Prior studies have shown ribotype diversity to be associated with geography, (5, 6), and with healthcare-or community origin (7, 8). Therefore, phylogenetic lineage assignment is useful for implicating CDI provenance, and identifying potential clonal expansion of *C. difficile* strains that could be indicative of disease outbreaks in healthcare settings.

There have been many CDI surveillance studies, but few have tracked ribotype diversity over extended periods. Such studies may be beneficial - in the United Kingdom, implementation of ribotype-based surveillance revealed lower than expected CDI incidence as well as a significant decrease in RT027 frequency (9, 10). The CDC’s “Emerging Infections Program – *Clostridioides difficile* infection surveillance” revealed a similar decrease in RT027 prevalence in the United States, concomitant with the expansion of RT106, which is now one of the most prevalent ribotypes (10, 11). In general, ribotype diversity can be extremely dynamic (12, 13), and the emergence of a ribotype in local, or national, contexts can be missed without longitudinal surveillance. In this study, we performed CDI surveillance and ribotyped recovered isolates over an 8-year period in a single tertiary healthcare setting with multiple in-patient hospital campuses and outpatient clinics. Our study specifically focused on clinical microbiology laboratory findings of CDI based on an enzyme immunoassay diagnostic protocol.

## Methods

### Specimen acquisition

Diarrheic stool specimens from patients with clinical suspicion of CDI were obtained over an eight-year period (2015–2022) from a > 600-bed tertiary Medical Center in Southern Arizona. All specimens were designated “to-be-discarded,” de-identified, and frozen by the Microbiology Laboratory. This study was deemed not to constitute human subjects research (NRDUC 1707612129) and was thus exempt from full Institutional Review Board approval. These specimens were subjected to a two-step diagnostic procedure that utilized a glutamate dehydrogenase-specific enzyme immunoassay (GDH-EIA) and a dual TcdA/TcdB-specific EIA (toxin EIA).

### Recovery and identification of *Clostridioides difficile*

The stool specimens were thawed, and an aliquot was plated onto taurocholate-cefoxitin-cycloserine-fructose agar (TCCFA) in an anaerobic chamber (Coy Laboratory Products, Grass Lake, MI, United States), and incubated at 37°C for 48 h. A colony from the TCCFA plate was re-struck to isolation on a new TCCFA plate and grown under the same conditions for 24 h. An isolated colony from the second TCCFA plate was propagated on Brain-Heart Infusion (BHI) agar and incubated at 37°C anaerobically for 24 h. From this plate, 1–3 colonies were used to inoculate 5 mL of BHI broth and incubated anaerobically overnight. The remaining bacteria from the BHI plate were cryopreserved in 25% glycerol in BHI and stored at –80°C.

Five ml of the overnight culture was centrifuged and resuspended in 1 mL of Tris-EDTA buffer and processed for genomic DNA extraction and ribotyping by the University of Arizona Genetics Core. Ribotyping PCR products were separated by capillary electrophoresis, and the lineage was identified by comparing the resulting electropherogram to Webribo, an international *C. difficile* ribotyping database (14). Ribotype assignment was considered ‘high-confidence’ based on the distance value ≤5 (14, 15).

### Toxin quantitation

A previously published method was used to quantify toxin production from *C. difficile* isolates (16). Briefly, *C. difficile* strains were grown in 5 mL BHI broth for 72 h. The cultures were then centrifuged, and cell-free supernatants clarified using 0.22 μm filters (Argos Technologies, Elgin, IL, United States). Toxin amounts in 50 μL of the supernatants were quantified using the Techlab^®^ *C. difficile* Tox A/B II™ EIA kit (Techlab, Blacksburg, VA, United States) per manufacturer’s instructions, with absorbance measured at 450 nm. Total protein concentration was quantified using the Pierce™ BCA Protein Assay Kit (ThermoFisher Scientific, Waltham, MA, United States), and toxin levels reported as absorbance at 450 nm/mg total protein. All data were collected in biological triplicate. Toxin status of the isolates was derived from the genotype (i.e., inferred from ribotype) or actual quantitation of toxin.

### Data analysis and visualization

Data analysis was performed and visualized in R Studios utilizing the tidyverse, dplyr, lucid and treemapify libraries. XLSTAT was used for statistical analyses. Student’s *t*-test, Mann–Whitney, and ANOVA were used to determine statistical differences between groups. Linear regression and goodness-of-fit was used to determine annual trends. A *p* value <0.05 was considered the threshold for significance. Linear regression was used to determine trends in annual ribotype frequency.

## Results

**Figure 1:**
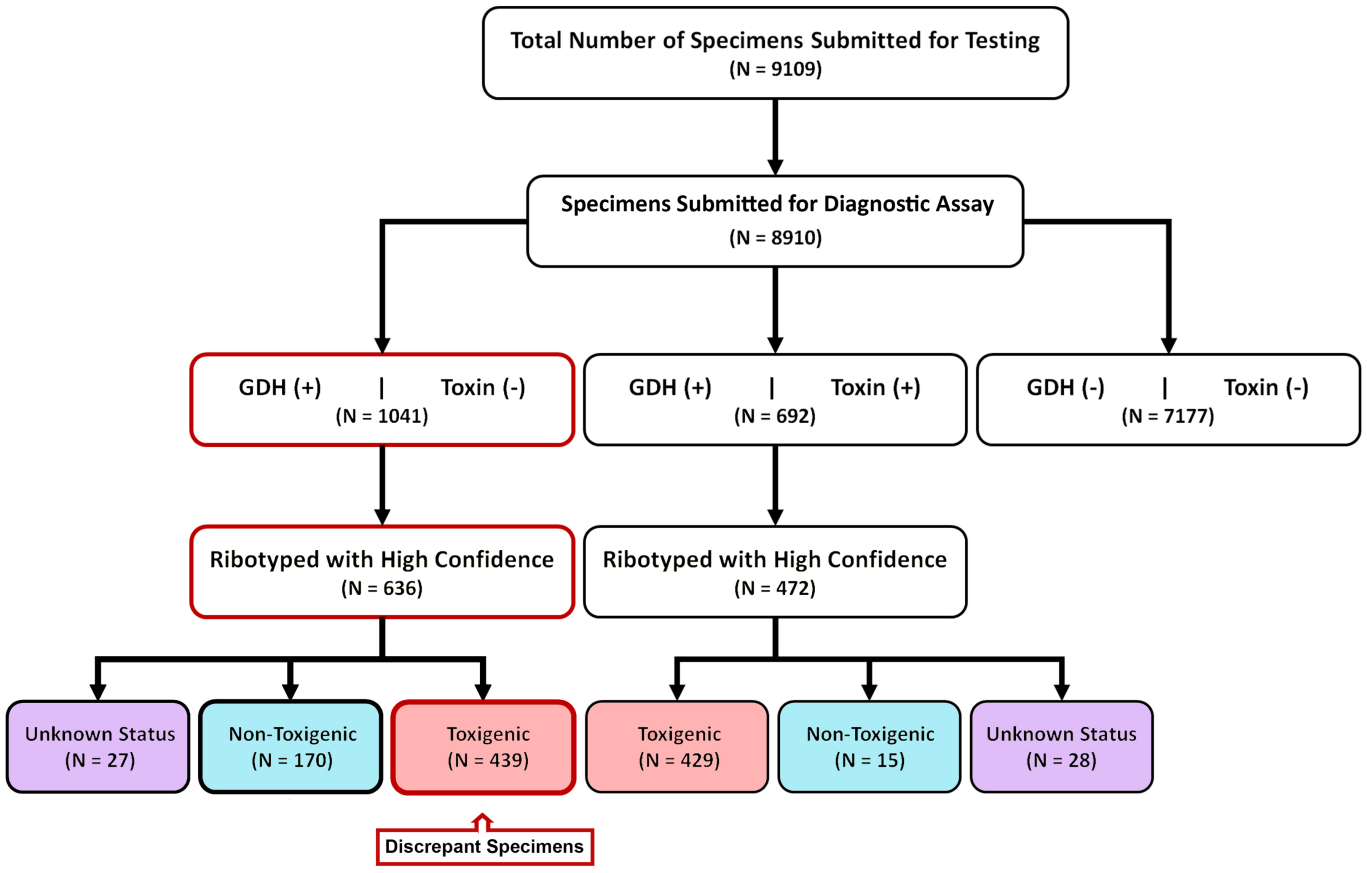
Study design and diagnostic results. From 2015–2022, 8,910 samples were diagnostically assayed for *C. difficile* utilizing a GDH-EIA and TcdA/B-EIA. 692 specimens were positive for both EIA (GDH+/Toxin+) and 1,041 specimens were GDH+ but Toxin–. *C. difficile* was isolated from 636 of 1,041 GDH+/Toxin– specimens and ribotyped. 170 specimens harbored non-toxigenic *C. difficile,* and 439 specimens harbored toxigenic *C. difficile*. These 439 specimens are considered discrepant, highlighted in red. Of the 692 GDH+/Toxin+ specimens, *C. difficile* isolates were isolated from 472 and ribotyped. 429 specimens (90.9%) harbored toxigenic *C. difficile*, and 15 specimens (3.2%) harbored non-toxigenic *C. difficile.*

9,109 diarrheic stool samples were submitted for *C. difficile* diagnostic testing over an eight-year period (2015–2022) (Figure 1). These specimens were received from 106 units in the Medical Center spanning 5 inpatient and outpatient campuses. 8,910/9109 specimens were subjected to a two-step diagnostic procedure that utilized a glutamate dehydrogenase-specific enzyme immunoassay (GDH-EIA) and a dual TcdA/TcdB-specific EIA (toxin EIA). *C. difficile* was recovered from 1733 specimens for a raw overall specimen positivity of 19.45%. Overall, 692/1733 specimens (39.9%) had detectable stool toxin (GDH+/Toxin+) and 1041/1733 specimens (60.1%) were stool toxin-negative (GDH+/Toxin–; Figure 1). *C. difficile* recovery was attempted from all clinical specimens, with at least one unique isolate per specimen cup. Ribotype designation was used to infer toxigenic potential since there is an association with ribotype and toxigenic status (17). Only high-confidence ribotype assignments were included in this study.

Of the 692 GDH+/Toxin+ specimens, *C. difficile* was successfully recovered from 472 individual sample containers. This recovery rate is similar to previously published reports, and inability to recover the bacterium from the remaining specimens may reflect absence of viable spores/vegetative cells. Using ribotype-based inference, 90.9% of these strains (429/472) were classified as toxigenic *C. difficile*. A small number of isolates (15/472; 3.2%) were classified as non-toxigenic. The clinical test-positivity of their corresponding specimens could be due to a false-positive EIA test result, or a co-occurring toxigenic *C. difficile* strain(s); this was not further investigated. Ribotypes could not be reliably assigned for 28 isolates.

For the 1,041 GDH+/Toxin– specimens, *C. difficile* was recovered from 636 individual containers. Based on ribotype inference, 26.7% (170/636) strains were non-toxigenic *C. difficile*. However, 69% (439/636) strains were inferred to be toxigenic. Since these 439 toxigenic isolates were recovered from specimens testing negative on a toxin EIA, we deemed those specimens to be “discrepant” relevant to the diagnostic paradigm. Ribotypes could not be reliably assigned for 27 isolates (Figure 1).

**Figure 2:**
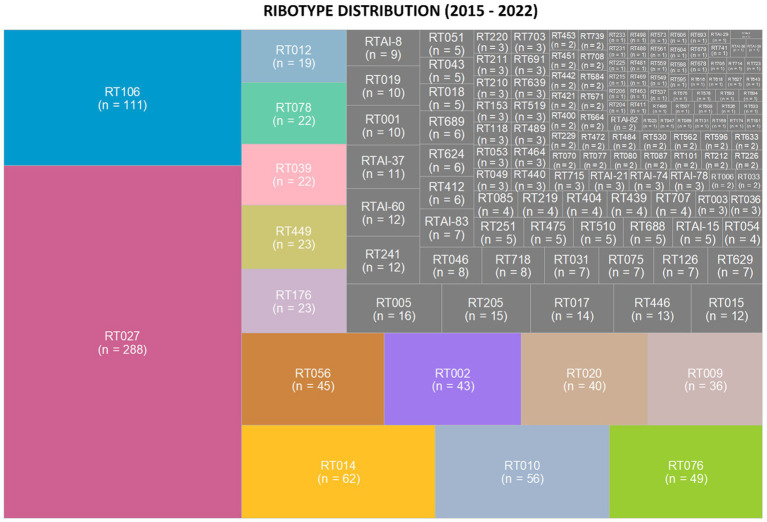
*Clostridioides difficile* ribotype distribution during an 8-year prospective surveillance (2015–2022). Tree map of the most prevalent ribotypes, in color, where the area of the box corresponds to the n-value. RT027 and RT106 were consistently the most common ribotypes per year. The non-toxigenic ribotypes RT010 and RT009 were the fourth and ninth most common ribotypes isolated from patient specimens.

**Figure 3:**
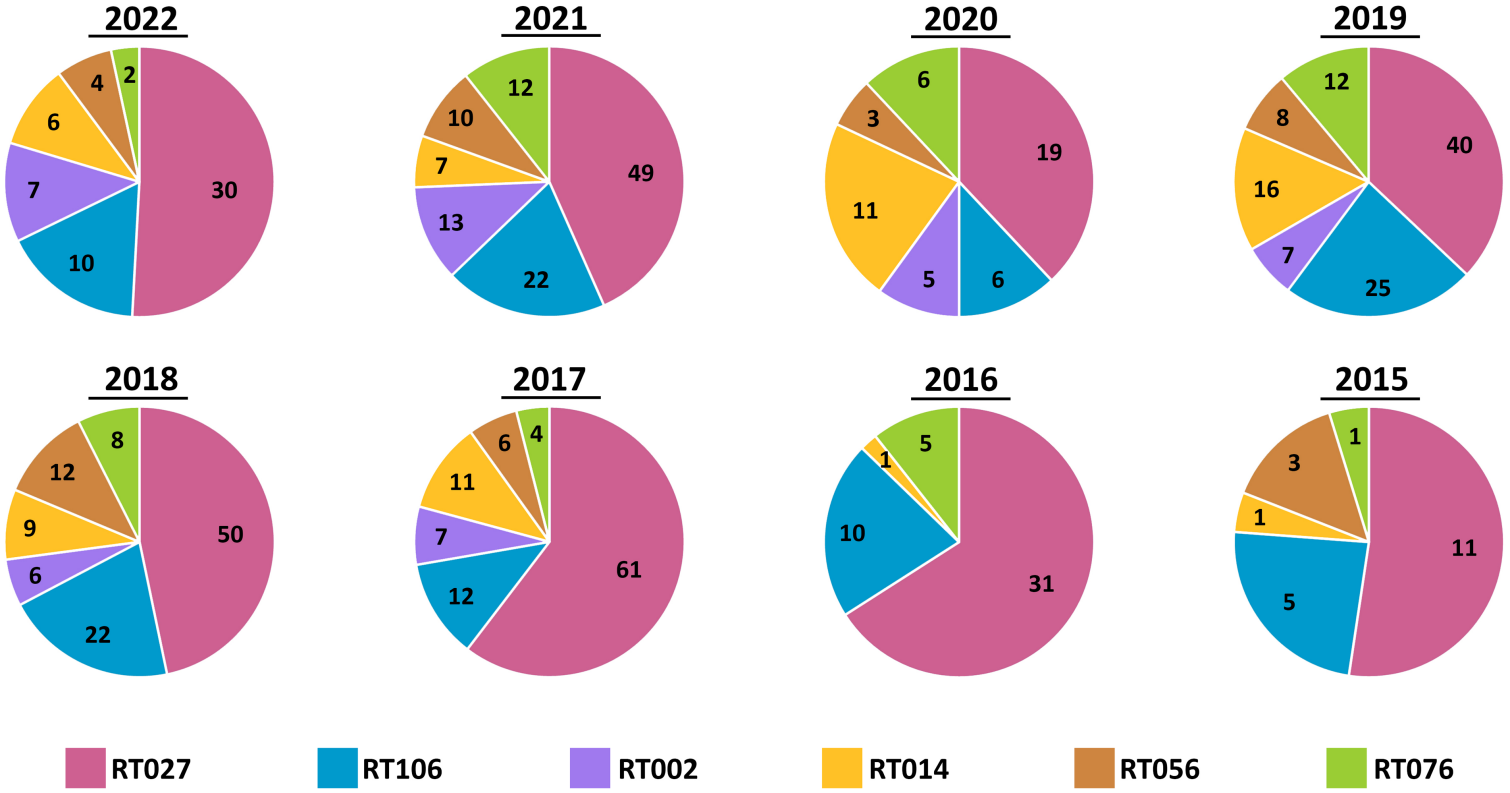
Annual distribution of most common *C. difficile* ribotypes is largely invariant. Pie charts showing the prevalence of the six most common ribotypes (RT027, RT106, RT002, RT014, RT056, and RT076) in totality, per year. While the number of cases varied by year, the relative frequencies did not vary. RT027 was consistently the most common ribotype isolated each year and RT106 was the second-most common ribotype, with the exception of 2020.

Ribotypes isolated from each specimen are reported in total (Figure 2) as well as *per annum* (Figure 3). The six most prevalent ribotypes isolated over the eight-year period (2015–2022), in descending order, are RT027 (*n* = 288), RT106 (*n* = 111), RT014 (*n* = 62), RT010 (*n* = 56), RT076 (*n* = 49), and RT056 (*n* = 45). When comparing the frequency of these ribotypes annually, the pattern remains largely unchanged from year to year (Figure 3). RT027 was the most prevalent ribotype isolated when a specimen was *C. difficile*-positive, with RT106, typically, being the second most prevalent (Figure 3). Importantly, during the entire surveillance period, and based on the observation that specimens from units reporting multiple CDIs always yielded isolates of non-overlapping ribotypes, we concluded that no major CDI outbreaks occurred in our healthcare facility.

**Figure 4:**
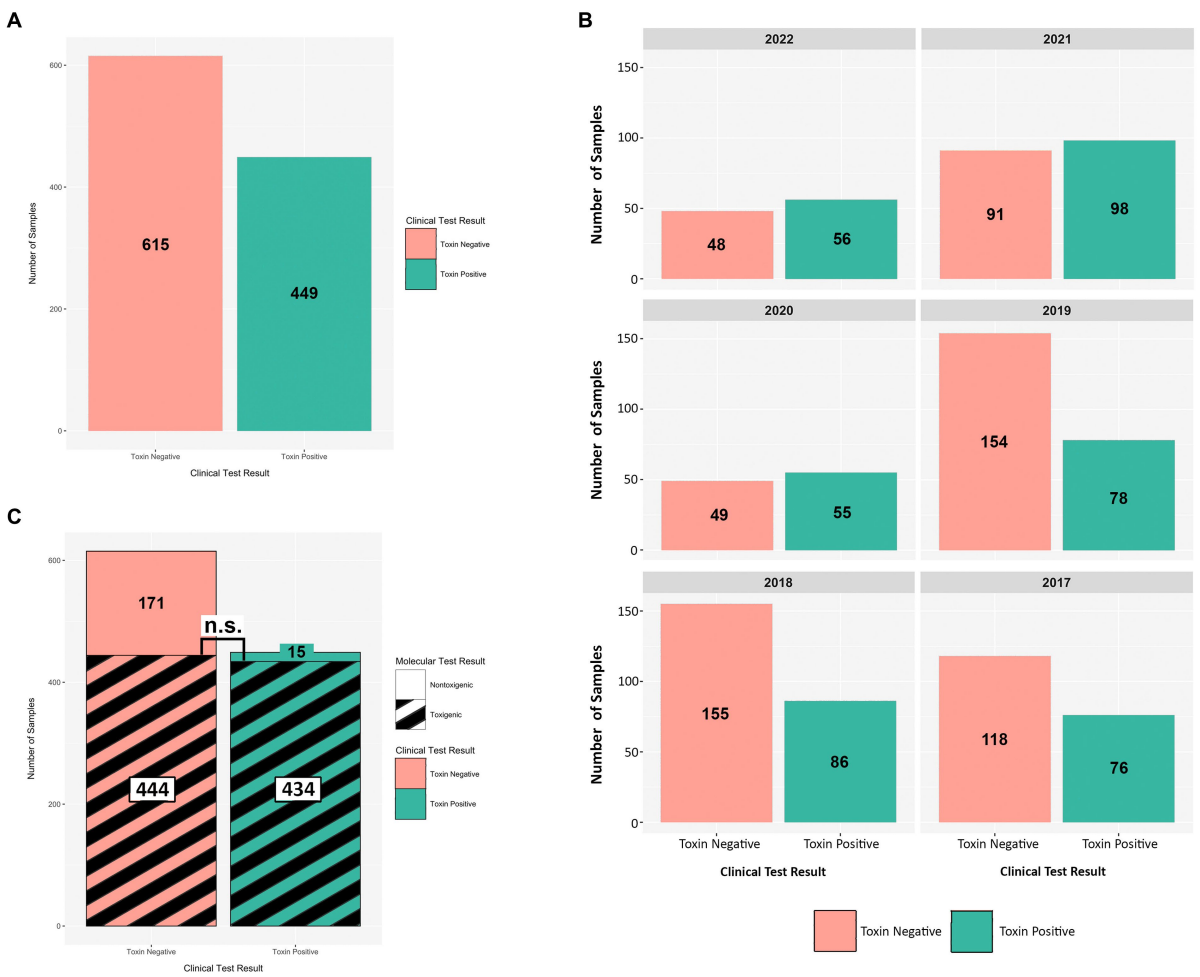
Toxin EIA results do not predict *C. difficile* toxigenic potential. Comparisons of toxin EIA results and strain toxigenicity as inferred by ribotype. **(A)** Toxin EIA-negative (peach) specimens were more numerous than toxin EIA-positive (green) specimens (615 vs. 449, respectively). **(B)** Annual distribution of specimens by toxin EIA result. **(C)** Of the toxin EIA-positive specimens, 434/449 (96.7%) harbored toxigenic *C. difficile* with 15/449 (3.3%) harboring non-toxigenic ribotypes. For the toxin EIA-negative specimens, 171/615 (27.8%) harbored non-toxigenic *C. difficile* but 444/615 (72.2%) specimens harbored toxigenic *C. difficile.* There were more non-toxigenic strains in toxin EIA-negative specimens but there were similar numbers of toxigenic *C. difficile* isolated from both groups (n.s. *p* > 0.4; Mann–Whitney).

From 2017 onward, a *C. difficile* testing policy was instituted wherein the GDH EIA and toxin EIA tests were employed as primary diagnostic assays. During this period (2017–2022), we received a total of 1,064 diarrheic GDH+ specimens of which 449 were toxin EIA-positive, and 615 were toxin EIA-negative (Figure 4A). Throughout the study period, the prevalence of the GDH+/Toxin– specimens was either similar to (2020–2022), or greater than (2017–2019), that of GDH+/Toxin+ specimens (Figure 4B). Overall, we recovered 449 unique *C. difficile* isolates from GDH+/Toxin+ specimens, with 449 (96.7%) inferred as toxigenic via ribotyping. Strikingly, 444/615 isolates from GDH+/Toxin-specimens (72.2%) were also inferred as toxigenic. In a head-to-head comparison, there was no statistical difference between the prevalence of toxigenic strains recovered from toxin EIA-positive and toxin EIA-negative specimens (Figure 4C; hatched black lines).

**Figure 5:**
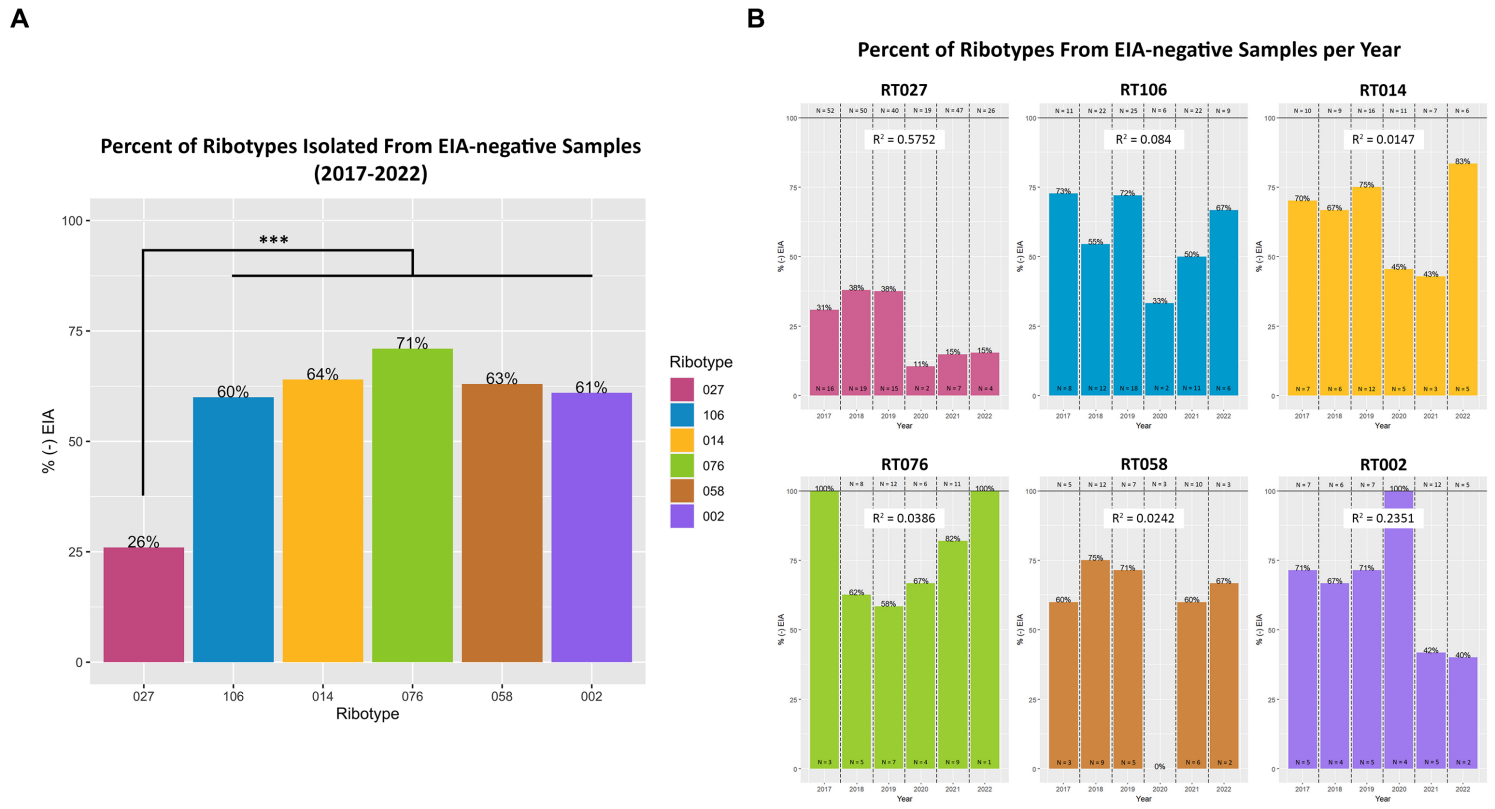
Toxigenic *C. difficile* ribotypes are isolated from toxin EIA-negative specimens. **(A)** The percentage of toxin EIA-negative strains, compared to the total number of strains, of the six most prevalent toxigenic ribotypes. Percentage values noted at top of bar. RT027 was significantly less prevalent in toxin EIA-negative specimens compared to the other five ribotypes (^***^p < 0.001; ANOVA). However, 26% of RT027 isolates, and > 60% of other ribotypes, were isolated from GDH+/Toxin– specimens. **(B)** Annual frequency of the 6 most common ribotypes, isolated from GDH+/Toxin– specimens. Goodness-of-fit (*R*^2^) of a linear regression reported for each ribotype.

Next, we set out to establish whether specific toxigenic *C. difficile* lineages were more likely to be classified as GDH+/Toxin– during clinical stool testing (i.e., more likely to be ‘discrepant’). Amongst the GDH+/Toxin-specimens, RT027 isolates were the least prevalent (26%), as compared with RT106 (60%), RT104 (64%), RT076 (71%), RT058 (63%) and RT002 (61%) (Figure 5A). The most prevalent ribotypes in this study, with the exception of RT027, were more likely to be isolated from toxin EIA-negative specimens than toxin EIA-positive specimens. While these frequencies did vary year to year, no significant trend in frequency changes was observed (Figure 5B).

**Figure 6:**
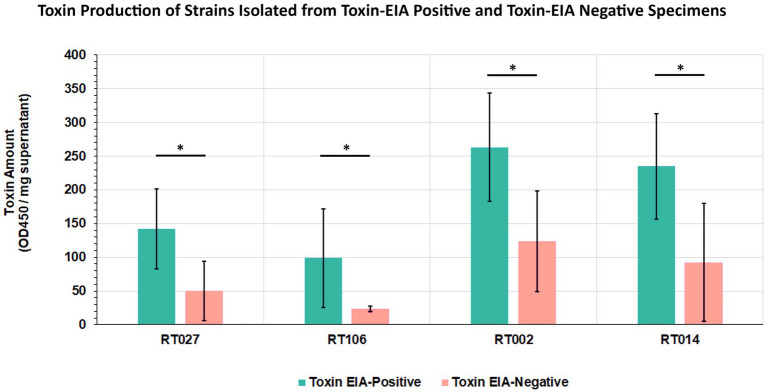
*Clostridioides difficile* from discrepant and non-discrepant specimens produce the major intoxicants TcdA/B. Five strains from toxin EIA-positive and toxin EIA-negative specimens each, belonging to four ribotypes (RT027, RT106, RT002, RT014), were assayed for toxin production. Bars represent average toxin produced (OD_450nm_) per mg of protein. Irrespective of ribotype, all strains produced *C. difficile* toxin A/B; however, strains isolated from discrepant specimens produced less toxin compared to strains from non-discrepant, toxin EIA-positive specimens. (^*^p < 0.05; *t*-test).

Given that all our samples were from diarrheic patients, we hypothesized that GDH+/Tox-specimens harbored toxigenic *C. difficile* that expresses intoxicants at levels lower than those detectable by the clinical toxin EIA. To assess this, we isolated *C. difficile* from a subset of specimens spanning the 4 most common ribotypes and used EIA to quantitate toxin abundance in bacterial culture supernatants. Therefore, 5 unique isolates from each of ribotypes RT027, RT106, RT002, and RT014 recovered from toxin EIA-negative specimens were compared with 5 unique isolates from each of the ribotypes above, but recovered from toxin EIA-positive specimens (Figure 6). Overall, all isolates from GDH+/Toxin– specimens indeed produced measurable toxin amounts, but close to the limit of detection of the clinical EIA. On average, these isolates produced less toxin than their counterparts of the same ribotype recovered from GDH+/Toxin+ specimens.

## Discussion

The CDC’s Emerging Infections Program (EIP) as well as various US healthcare facilities track and stratify CDI cases as having healthcare-or community-onset. CDI is confirmed by detecting TcdA and/or TcdB in stool specimens using an EIA or a molecular assay such as a nucleic acid amplification test (NAAT/PCR) for *tcdB.* Binary toxin (CDT) is not utilized for diagnostic purposes as the contribution of CDT to pathogenesis is not clear and the genes are not uniformly present in *C. difficile* (18, 19). These approaches allow for prospective and retrospective investigations wherein CDI prevalence can be established with no additional testing. However, CDI surveillance efforts do not routinely track ribotypes or other molecular phylogeny. We posit that such efforts, particularly ribotype identification, can provide an early alert of potential outbreaks, define regional prevalence, track dispersal, and monitor disease manifestation/association and treatment response(s) (4, 6, 20–24).

Few CDI surveillance studies have reported longitudinal ribotype distributions, and the CDC has not reported U.S. national *C. difficile* ribotype prevalence since 2018 (25). In their decade-long prospective surveillance in Dublin, Skally et al. noted that the most prevalent ribotypes, leading with RT014 and RT002, did not change annually (26). Our study also reveals stable distribution of ribotypes in our patient population/healthcare system over the eight-year surveillance period. This invariance in ribotype distribution provides additional support for the technique’s usefulness in identifying potential outbreaks, since unexpected changes in *C. difficile* phylogenetic lineages can be easily detected. Indeed, Skally et al. identified outbreaks by detecting the emergence of new ribotypes or the altered frequency of existing ribotypes (26).

While largely invariant locally, there are geographical differences in ribotype distribution that may be masked by national aggregate data (27). The CDC EIP for *C. difficile* only tracks cases from 10 select states in the U.S., and the reported ribotypes may not accurately reflect regional observations in the rest of the country. For example, a six-center surveillance study encompassing the states of Illinois, Minnesota, New York, Montana, Massachusetts, and California (*N* = 939), as well as a Texas surveillance study (*N* = 3,877) both reported RT078 and RT255, respectively, as their most prevalent ribotypes (13, 28). There was no geographical overlap between these studies and the CDC EIP which did not include these two lineages in the report’s 10 most-prevalent ribotypes list (11). Such findings underscore the importance of regional ribotyping efforts in addition to those that report nationwide aggregates.

GDH+ stool specimens may be negative for the toxin EIA test either because they harbor non-toxigenic *C. difficile* strains, or because the amount of toxin in the stool sample is below the threshold of detection in the clinical test. We have demonstrated that *C. difficile* isolated from a subset of the latter group also produce lower levels of toxin during *in vitro* culture and also in the rodent intestine, relative to ribotype-matched isolates from GDH + Toxin+ specimens (16). Nevertheless, such low-toxin *C. difficile* strains exhibited comparable virulence to high-toxin strains in a hamster model of acute CDI (16).

From a clinical perspective, the significance and impact of diagnostically-discrepant *C. difficile* continues to be debated (29–32). The relative lower sensitivity of stool toxin EIA relative to NAAT has been leveraged as a benefit in discriminating active CDI from situations where *C. difficile* colonization is considered incidental (even if the bacteria encode the toxins), or a minor contributor to disease symptoms. However, Anikst et al., and others, have reported that organism burden and stool toxin levels were not significantly different between patients with and without severe diarrhea (30, 33–35). Since CDI affects patients asymmetrically, and since diagnostically toxin-negative specimens can harbor virulent *C. difficile* (24, 36–38), a deeper assessment of pathogen phylogeny may be valuable. Currently, the presence of particular ribotypes (e.g., RT027) can be considered a predictor for severe disease and recurrent CDI (39, 40) though others indicated otherwise (41). Large-scale surveillance studies that correlate ribotypes, among other factors, with patient outcomes will be able to clarify this discordance.

Since reflex testing (to a NAAT) is generally not recommended (42), the decision for CDI treatment when stool specimens are GDH + Toxin-is delegated to clinician judgement (1). Concerningly, Reigadas et al. reported that 12.7% of *C. difficile* infection cases would be missed due to a lack of significant clinical suspicion (43). A lack of clinical suspicion was previously shown to account for a three-fold increase in CDI misdiagnosis in a meta-analysis of hospitals across 12 European countries (44). Skally et al. also noted that if a universal testing scheme had not been adopted, most CDI cases in their surveillance would have been undiagnosed (26). Mawer et al. further reported that patients with diarrhea, and harboring toxigenic *C. difficile*, but testing GDH+/Toxin-, contributed to ≥25% of in-hospital CDI transmission (32).

Our current study, and other reports, highlight the potential scale of this diagnostic gray area. Our findings indicate that up to 69% of the GDH+/Toxin-specimens nevertheless harbor toxigenic *C. difficile*. Similarly, Akamatsu et al. reported toxigenic *C. difficile* to be present in 63.2% of GDH+/Toxin– specimens (45). The CDC estimates that discrepant specimens (NAAT+/Toxin–) constitute 31% of the national sampling set (25), although regional differences may be masked in national averages. Since we recovered low-toxin strains from the four most prevalent ribotypes also identified as USA-dominant in the CDC’s EIP *C. difficile* surveillance (11), the need for heightened clinical awareness regarding a GDH+/TOX-test result is warranted.

A particular strength of our study is the sample size since specimens were collected throughout an 8-year period. These numbers, and an unbiased ribotyping scheme, allowed us to determine relative phylogenetic lineage variance on a year-to-year, even month-to-month, basis. To our knowledge, our study is the first to elucidate the ribotype distribution of discrepant CDI-suspicion stool specimens. Except for RT027, the most prevalent USA ribotypes are more likely to be isolated from GDH+/Toxin– specimens than GDH+/Toxin+ specimens. This is especially notable for rapidly-expanding ribotypes such as RT106, since they can be extremely virulent despite producing low amounts of toxin (15, 16).

Our study does have some limitations. Foremost is that 1–3 *C. difficile* colonies were used (per specimen), to assign ribotype. As such, we cannot rule out the possibility of co-occurrence of multiple *C. difficile* ribotypes in the original specimens. Previously published work estimates that 5–10% of CDI specimens may harbor more than one strain (46) but more recent work suggest this to be the case only in 1.5% of the specimens (47). Another limitation is that our specimens were obtained from a single healthcare center (albeit with over 106 units and 5 inpatient and outpatient campuses); specimens from other sites are necessary to generalize our local ribotype distribution. Finally, no patient correlates were analyzed during this surveillance. Thus, despite hospital Unit and collection dates being unique throughout the study, we do not know how many specimens were from repeat testing of the same patient. Correlating patient outcomes, including disease severity, progression and recurrence, to ribotype and diagnostic test results will be a focus in future efforts.

Overall, this study robustly demonstrates the benefit of prospective CDI surveillance. Our findings indicate that toxigenic *C. difficile* strains, including those belonging to outbreak-associated ribotypes, are frequently isolated from toxin-negative specimens. A modulated diagnostic paradigm, especially one that includes some unbiased phylogeny, may be necessary in the future to accurately detect toxigenic *C. difficile* and minimize clinical uncertainty.

## Data availability statement

The raw data supporting the conclusions of this article will be made available by the authors, without undue reservation.

## Author contributions

FA: conceptualization, methodology, investigation, analysis, writing original draft, and review and editing. MC: conceptualization, investigation, analysis, and review and editing. JL, RC-W, AS, EN, and JB: investigation and analysis. WL and KS: resources and review and editing. VV and GV: conceptualization, funding acquisition, project administration, supervision, and review and editing. All authors contributed to the article and approved the submitted version.
